# Supplementary material for: Causal Relationship between Aging and Anorexia Nervosa: A White-Matter-Microstructure-Mediated Mendelian Randomization Analysis
Source: Biomedicines. 2024 Aug 16;12(8):1874. doi: 10.3390/biomedicines12081874 (PMC11351342; doi:10.3390/biomedicines12081874)
Supplement: Supplementary file 1 [file biomedicines-12-01874-s001.zip › Supplementary Figure/Figure S4/Singe SNP plot of MR analysis for TL to ubm-b-1878.pdf]

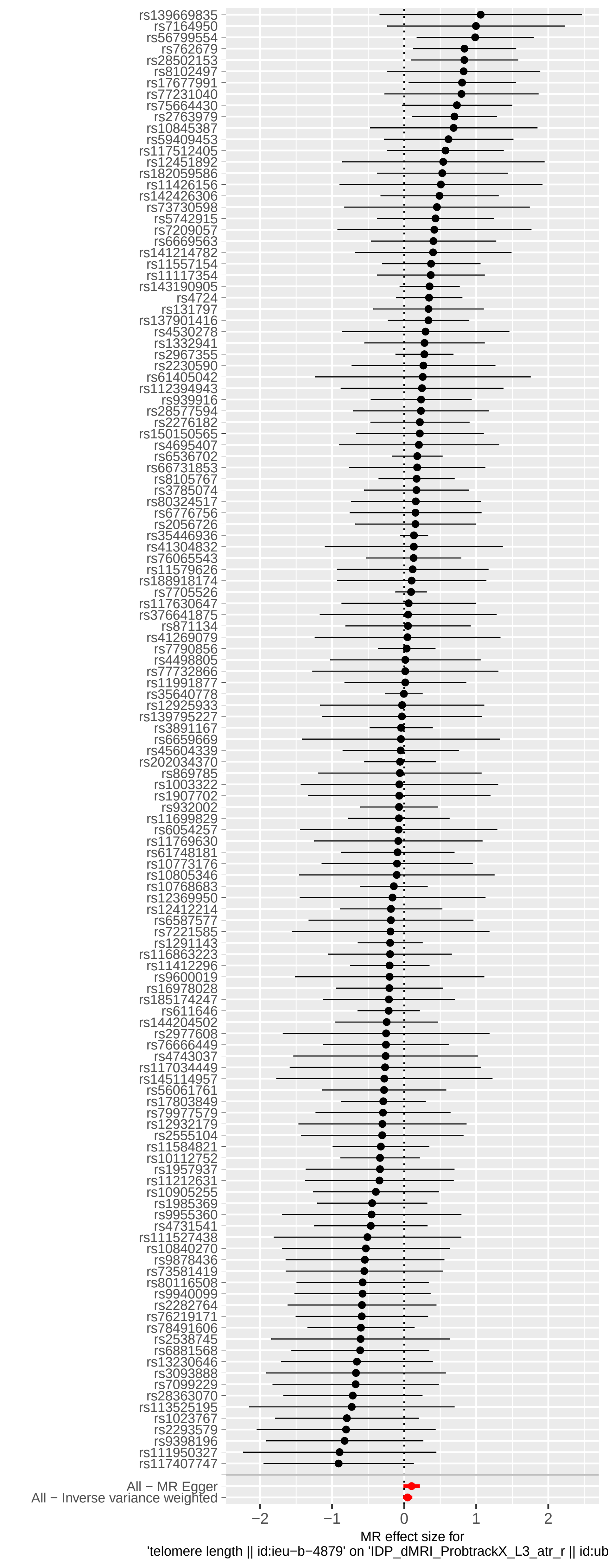
rs139669835
rs7164950
rs56799554
rs762679
rs28502153
rs8102497
rs17677991
rs77231040
rs75664430
rs2763979
rs10845387
rs59409453
rs117512405
rs12451892
rs182059586
rs11426156
rs142426306
rs73730598
rs5742915
rs7209057
rs6669563
rs141214782
rs11557154
rs11117354
rs143190905
rs4724
rs131797
rs137901416
rs4530278
rs1332941
rs2967355
rs2230590
rs61405042
rs112394943
rs939916
rs28577594
rs2276182
rs150150565
rs4695407
rs6536702
rs66731853
rs8105767
rs3785074
rs80324517
rs6776756
rs2056726
rs35446936
rs41304832
rs76065543
rs11579626
rs188918174
rs7705526
rs117630647
rs376641875
rs871134
rs41269079
rs7790856
rs4498805
rs77732866
rs11991877
rs35640778
rs12925933
rs139795227
rs3891167
rs6659669
rs45604339
rs202034370
rs869785
rs1003322
rs1907702
rs932002
rs11699829
rs6054257
rs11769630
rs61748181
rs10773176
rs10805346
rs10768683
rs12369950
rs12412214
rs6587577
rs7221585
rs1291143
rs116863223
rs11412296
rs9600019
rs16978028
rs185174247
rs611646
rs144204502
rs2977608
rs76666449
rs4743037
rs117034449
rs145114957
rs56061761
rs17803849
rs79977579
rs12932179
rs2555104
rs11584821
rs10112752
rs1957937
rs11212631
rs10905255
rs1985369
rs9955360
rs4731541
rs111527438
rs10840270
rs9878436
rs73581419
rs80116508
rs9940099
rs2282764
rs76219171
rs78491606
rs2538745
rs6881568
rs13230646
rs3093888
rs7099229
rs28363070
rs113525195
rs1023767
rs2293579
rs9398196
rs111950327
rs117407747
All − MR Egger
All − Inverse variance weighted
−2
−1
0
1
2
MR effect size for
'telomere length || id:ieu−b−4879' on 'IDP_dMRI_ProbtrackX_L3_atr_r || id:ub
